# Depression predictions from GPS-based mobility do not generalize well to large demographically heterogeneous samples

**DOI:** 10.1038/s41598-021-93087-x

**Published:** 2021-07-07

**Authors:** Sandrine R. Müller, Xi (Leslie) Chen, Heinrich Peters, Augustin Chaintreau, Sandra C. Matz

**Affiliations:** 1 Data Science Institute, Columbia University, New York, USA; 2 Computer Science Department, Columbia University, New York, USA; 3 Columbia Business School, Columbia University, New York, USA; 4 Present Address: Department of Psychology, Bielefeld University, Bielefeld, Germany

**Keywords:** Human behaviour, Depression, Psychology and behaviour, Computational science

## Abstract

Depression is one of the most common mental health issues in the United States, affecting the lives of millions of people suffering from it as well as those close to them. Recent advances in research on mobile sensing technologies and machine learning have suggested that a person’s depression can be passively measured by observing patterns in people’s mobility behaviors. However, the majority of work in this area has relied on highly homogeneous samples, most frequently college students. In this study, we analyse over 57 million GPS data points to show that the same procedure that leads to high prediction accuracy in a homogeneous student sample (N = 57; AUC = 0.82), leads to accuracies only slightly higher than chance in a U.S.-wide sample that is heterogeneous in its socio-demographic composition as well as mobility patterns (N = 5,262; AUC = 0.57). This pattern holds across three different modelling approaches which consider both linear and non-linear relationships. Further analyses suggest that the prediction accuracy is low across different socio-demographic groups, and that training the models on more homogeneous subsamples does not substantially improve prediction accuracy. Overall, the findings highlight the challenge of applying mobility-based predictions of depression at scale.

## Introduction

Depression is a common mental health disorder—In the U.S. alone, an estimated 17.3 million of adults have experienced at least one major depressive episode^1^. At its worst, depression can lead to attempted suicide and the loss of a human life. The World Health Organization estimates that every year, nearly 800,000 people die from suicide^2^. What makes this statistic particularly upsetting is that, after decades of research, the medical community has found several effective treatments for depression. However, all too often the people who need these treatments the most are not properly diagnosed^3^.

Recent advances in mobile sensing technologies and machine learning have sparked hope and optimism among scientists that predictive modelling could revolutionize the way depression assessment is conducted. While traditional diagnostic assessments rely on in-person screening, and therefore require individuals to take the first step in seeking out medical advice, a growing body of literature suggests that a person’s propensity to develop depression can be passively measured by observing patterns in their mobility behaviors (see^4,5^ for a comprehensive review of the literature). In line with the current International Disease Classification’s (ICD-10) depression symptoms of loss of interests and fatigue^6^, people who suffer from depression, for example, have been found to move less and to be less likely to leave their home^7–9^.

Alongside the rapid accumulation of scientific studies supporting the diagnostic value of smartphone based metrics^10–14^, there has been a growing number of commercial and non-profit tracking applications aimed at putting this scientific evidence into practice (e.g. Mindstrong^15^, Ksana Health^16^). While the integration of such technologies into existing diagnostic settings has the potential to improve the early detection and treatment of depression, there is little scientific evidence to corroborate that such predictions can work at a general population level. In fact, the existing research in support of the predictive power of mobility has heavily relied on student samples, which are often highly homogeneous both in terms of their socio-demographic composition as well as their mobility patterns^9,12,17–22^.

If insights obtained from small and homogeneous samples are meant to be used in real-world applications targeted at the general population, it is paramount to establish the predictive performance of such models in the general population. Without proper validation, the implementation of smartphone-based diagnostic tools could, in fact, cause more harm than good. Inaccurate diagnostic output can entail unintended consequences in at least two ways: False negatives may prevent individuals from seeking out the right venues for further diagnostic assessment and treatment. False positives can cause sub-clinical individuals to seek help and bind scarce resources.

Making matters worse, the cost of inaccurate diagnostics is often distributed unequally between individuals. The predictive modelling literature offers numerous examples of how algorithmic tools can unintentionally discriminate against certain demographic groups, including those legally protected^23–30^. The algorithm may simply not “see” enough examples to extract and integrate patterns that might be specific to a particular sub-population^31^. For instance, middle-aged or elderly people, as well as individuals in sparsely populated rural areas, and less affluent communities may be underrepresented in samples collected using smartphone sensors. As a result, algorithmic diagnostics validated on population-level data may under-perform when applied to individuals with uncommon lifestyles, or in the worst case, systematically bias predictions such that they become less accurate than a random guess. That is, bias may be introduced that puts individuals with certain characteristics at a higher risk of misdiagnoses (either positive or negative) simply because rules learned on the majority generalize poorly to those individuals’ behavior.

Previous research has demonstrated high predictive performance on student samples which are highly homogeneous in terms of lifestyle and socio-demographic factors (e.g., being of a similar age, living in the same city, having similar daily schedules)^32,33^. Here, we study the generalizability of depression detection from GPS-based mobility data by scaling the approaches typically established in small, homogeneous samples to a large, heterogeneous sample of participants distributed across all fifty U.S. states and Washington D.C. (see Supplementary Fig. S2). Specifically, we investigate how accurately mobility predicts depression in a homogeneous student sample (N = 57) versus a heterogeneous population sample (N = 5262), using a variety of machine learning approaches (Research Question 1, RQ1). To further test the extent to which such algorithms might be systematically biased against certain subpopulations, we test how accurately a model trained on the general population predicts depression in different subpopulations (Research Question 2, RQ2). Finally, we examine whether the accuracy of predictions can be improved by training models separately for different subpopulations that are more homogeneous in their socio-demographic or mobility characteristics (e.g. 26–35 year old users living in an urban area; Research Question 3, RQ3).

## Results

The results are reported for two samples: (1) a homogeneous student sample collected on campus (“Students”; N = 57), and (2) a demographically and geographically heterogeneous U.S.-wide sample (“MindDoc users”; N = 5262). It is worth noting that the student sample is not included in the U.S.-wide MindDoc user sample. Both samples were collected through the self-tracking application MindDoc^34^, which allows users to continuously self-monitor their mood and depressive symptoms by responding to short surveys up to three times a day. Over the course of 14 days, the app then generated a validated depression assessment^35^. Moreover, participants provided informed consent to have their survey data matched against their GPS data and step count information (via the Google Fit API) via the MindDoc app. Three different machine learning algorithms—penalized logistic regression^36^, random forest^37^, and eXtreme Gradient Boosting (XGBoost)^38^—were used in conjunction with nested cross-validation to predict depressive symptoms in the two samples. Each model was trained as a classification task aimed at discriminating between participants with no depressive symptoms and participants with at least mild depressive symptoms (see “Methods” for more details).

### RQ1: How well does mobility predict depression in a homogeneous student versus a heterogeneous population sample?

**Figure 1:**
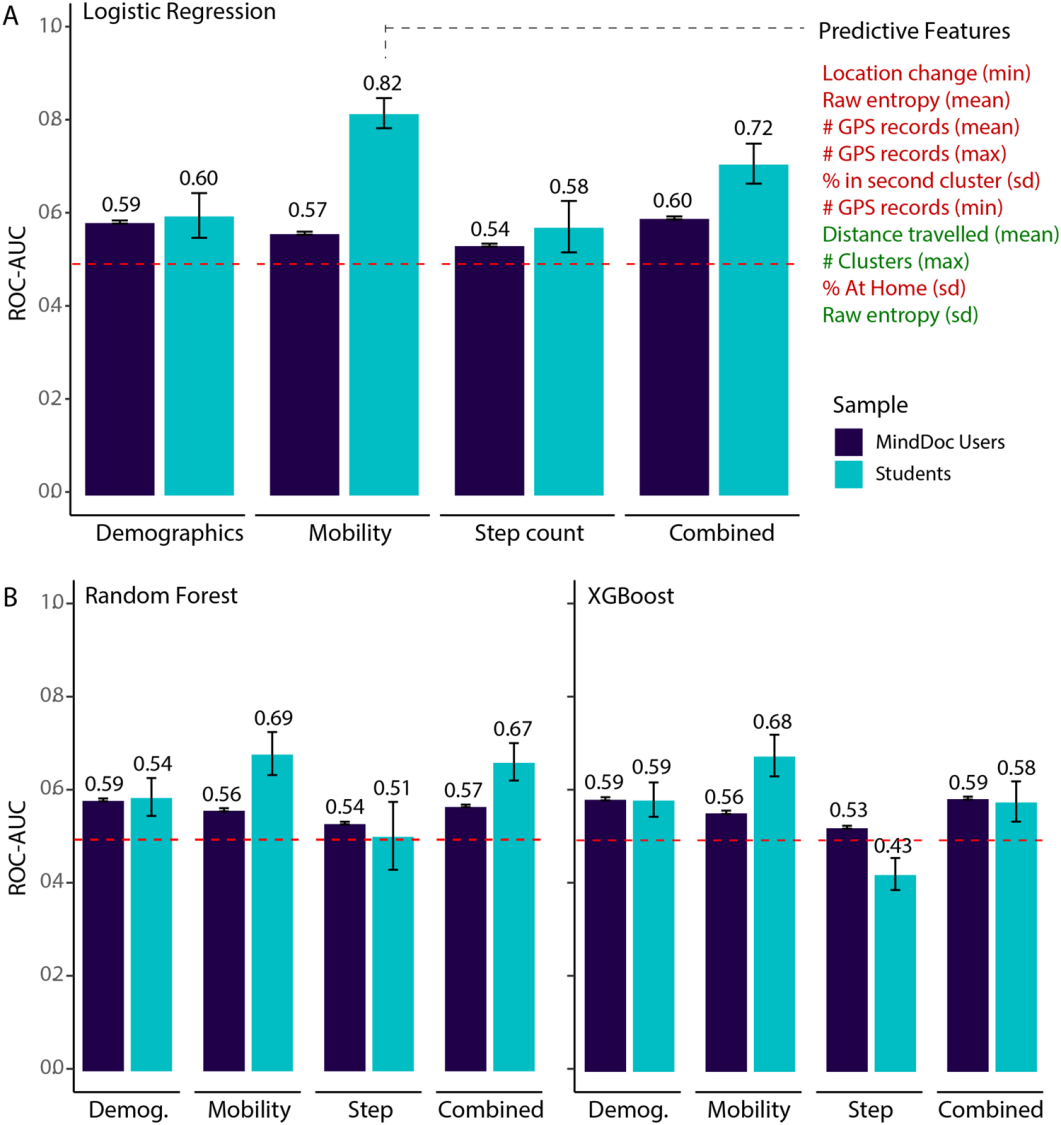
Predictive accuracies (AUC-ROC) and the 95% confidence intervals across the student and general population samples, using three different classifiers and four different feature sets. (**A**) Shows the results of logistic regression to predict depression from different data sources in college students (light blue) and the general population sample (dark blue). It also highlights the most predictive features in the mobility model for the student sample. Red (green) indicates a negative (positive) relationship with depression. (**B**) shows the corresponding results using random forest and XGBoost algorithms. Data collected using the MindDoc app (https://minddoc.de/app).

Among the three classifiers, penalized logistic regression was found to provide the highest predictive performance (area under the receiver-operating characteristic curve, AUC) across both samples (see Fig. 1A,B). The superior performance of logistic regression models compared to the more complex random forest and gradient boosting classifiers suggests that in our samples, variance in depression is best described as a linear function of predictors, and that adding more complex interactions between variables does not meaningfully improve predictive performance. In fact, for the relatively small student sample, the non-linear models seem to overfit and reduce—rather than increase—the out-of sample AUC compared to the simpler logistic regression. Consequently, we will focus our discussion of results on those obtained from the logistic regression models, and report the findings for the analyses associated with RQ2 and RQ3 for the logistic regression model only. In line with the results of previous studies, the AUC of depression prediction based on mobility data alone was found to be high in the student sample, with an AUC of $0.82\pm 0.03$ (see light blue bars in Fig. 1A). This means that when presented with a student who suffers from at least mild depression and a student who does not get classified as depressed, the algorithm correctly classifies the students in 82% of cases (an AUC of 0.5 is indicating the level of chance). Students with at least mild depression differed only slightly in their step patterns and demographic characteristics from those without (AUCs of $0.58\pm 0.06$ and $0.60\pm 0.05$, respectively). Adding demographic and step-based features to the mobility-based prediction model did not increase prediction performance (in fact, it led to a lower overall AUC score of $0.72\pm 0.04$ which might be indicative of overfitting). This finding suggests that the mobility behaviors capture the depression-related variance in the sample better than other traditional predictors of depression. The most predictive mobility features in the student sample were related to the number of GPS records, location changes, and entropy, such that students scoring higher on depression had a lower minimum number of location changes, had less GPS records overall, and showed lower entropy, i.e. greater inequality in the time spent in different places (see Fig. 1A).

Predictive performance in the U.S.-wide sample was found to be significantly lower compared to the student sample (mean = 0.82) (Mann–Whitney $U(N_{\text {MindDoc}}=100, N_{\text {Student}}=100) = 1156.0, p < 0.001 \text { two-tailed}$), and only slightly higher than chance ($\hbox {AUC} = 0.57\pm 0.003$, see dark blue bars in Fig. 1A). This finding suggests that, in more heterogeneous samples, participants with and without at least mild depression do not differ much in their mobility patterns. Although models trained on demographic variables showed similar performance compared to those observed in the student sample, predictive performance remained relatively low ($\hbox {AUC} = 0.59\pm 0.002$). Similar to the student sample, step count data provided the lowest predictive performance (AUCs of $0.54\pm 0.003$). Including all features in the prediction model only marginally increased prediction performance (AUC score of $0.60\pm 0.003$). Future work could further test this for additional subgroups based on other variables that have been previously linked to depression as well as mobility behaviors (e.g. physical disease, alcohol use^39–41^ ).

Taken together, it appears that while mobility patterns are predictive of depression in our homogeneous on-campus sample, they hold far less predictive power in a large, heterogeneous sample of U.S. users. This finding suggests that the current deployment of passive mood assessment apps and software, based on careful evaluation in homogeneous samples, might be misguided or would need to be complemented with other sensing signals to be truly effective at scale. Whereas mobility-based predictions of depression appear to provide high predictive performance in small samples that are homogeneous in their socio-demographic and mobility characteristics, they do not perform well in large, heterogeneous populations. We further explore these findings by breaking down the samples into more homogeneous subsamples (analogous to the student sample), to (1) establish whether the overall predictive performance is driven by weak predictive performance across all subpopulations or whether performance is unevenly distributed across subpopulations (RQ2), and (2) test whether the predictive performance could be improved by training models directly for these subsamples (RQ3).

### RQ2: How well does a model trained on the general population predict depression in different subpopulations?

**Figure 2:**
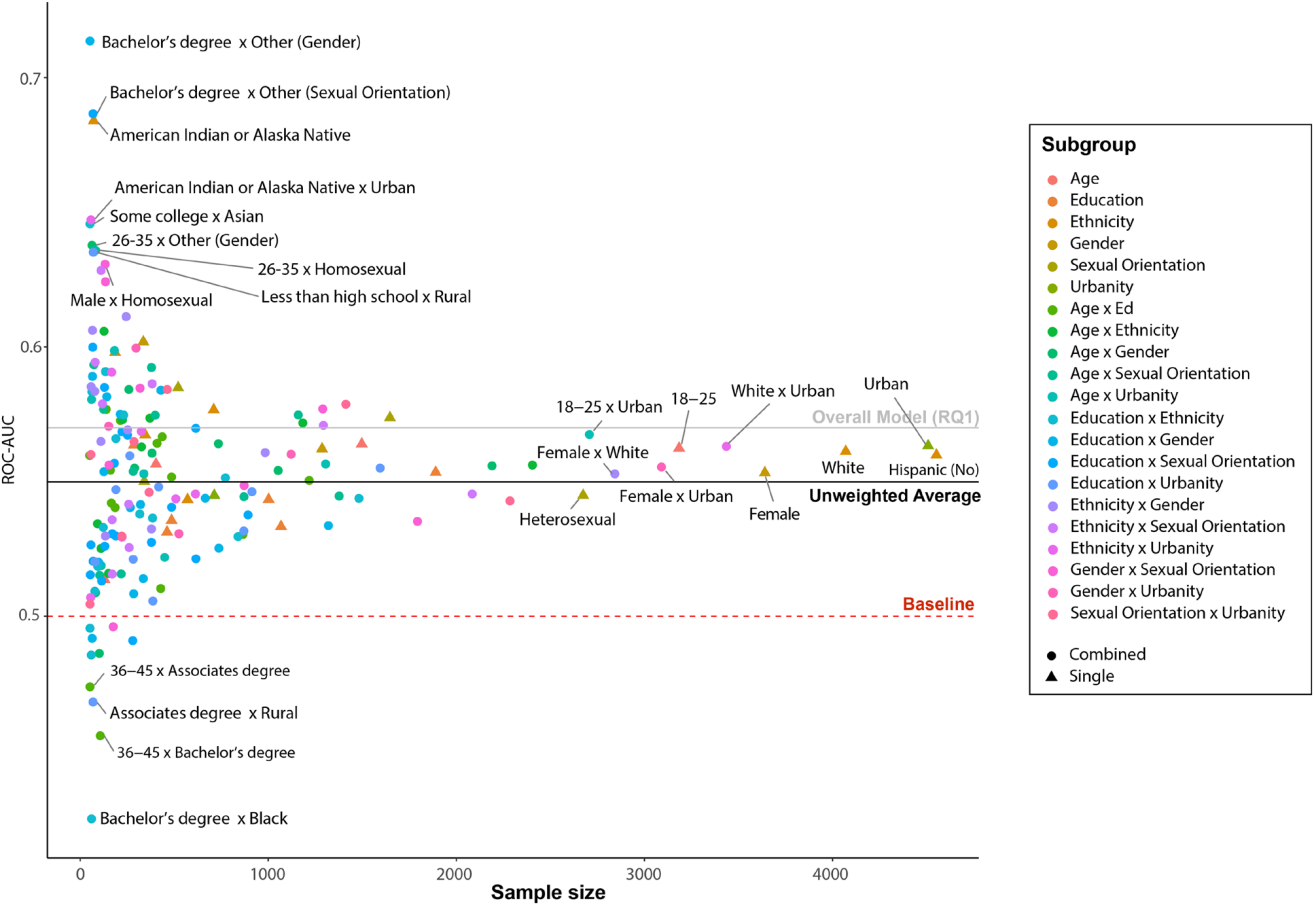
Average out-of-sample predictive performance (AUC) of different socio-demographic sub-population as a function of sample size. Shapes indicate whether the subpopulation is based on a single socio-demographic variable (triangles) or a combination of two variables (circles).

The predictive performance for specific subpopulations can depend on the representation of these subpopulations in the overall training sample. That is, training samples might not contain enough data on small minority groups for the algorithm to learn patterns specific to these subpopulations, leading to unintended discrimination. We therefore tested the extent to which our predictive models produced similar accuracy levels across different subgroups that were formed based on socio-demographic characteristics (e.g. all female participants, all participants aged 16–25, or all female participants aged 16–25). The subsamples included various levels for the following six socio-demographic characteristics as well as their two-way intersections: gender, age, education, urban/rural environment (as measured by the urban influence index^42^), sexual orientation and ethnicity. To increase the reliability of our findings, we only included subgroups with at least 50 individuals. If the algorithm did indeed discriminate against specific minority groups one would expect a particular set of characteristics clustered in the lower half of Fig. 2 (i.e. indicating low predictive performance) and a positive relationship between sample size and accuracy (i.e. with larger samples being better represented in the training set). However, as Fig. 2 shows, no such relationship was found in the current data (Pearson’s $r(195) = -0.003, p = 0.969$). Although the smaller groups on the left-hand side of the x-axis showed greater variance in predictive performance they did not show systematically lower performance than the larger groups on the right-hand side. We also did not observe any clear patterns suggesting that there are particular minority groups that show consistently low AUCs. For example, minority groups related to ethnicity, sexual orientation or education did not show consistently lower levels of predictive performance. The only group that appeared repeatedly among the lowest AUCs was the category “rural”. This suggests that the predictive performance of mobility-based depression detection might be lower for individuals living in rural areas, which likely see higher heterogeneity in the mobility behaviors of residents than those seen in urban areas. However, taken together, the analyses conducted in the context of RQ2 suggest that the predictive models did not introduce a strong, systematic bias against certain minority groups.

### RQ3: Can the predictive performance for the general population be improved by training models separately for different subpopulations?

**Figure 3:**
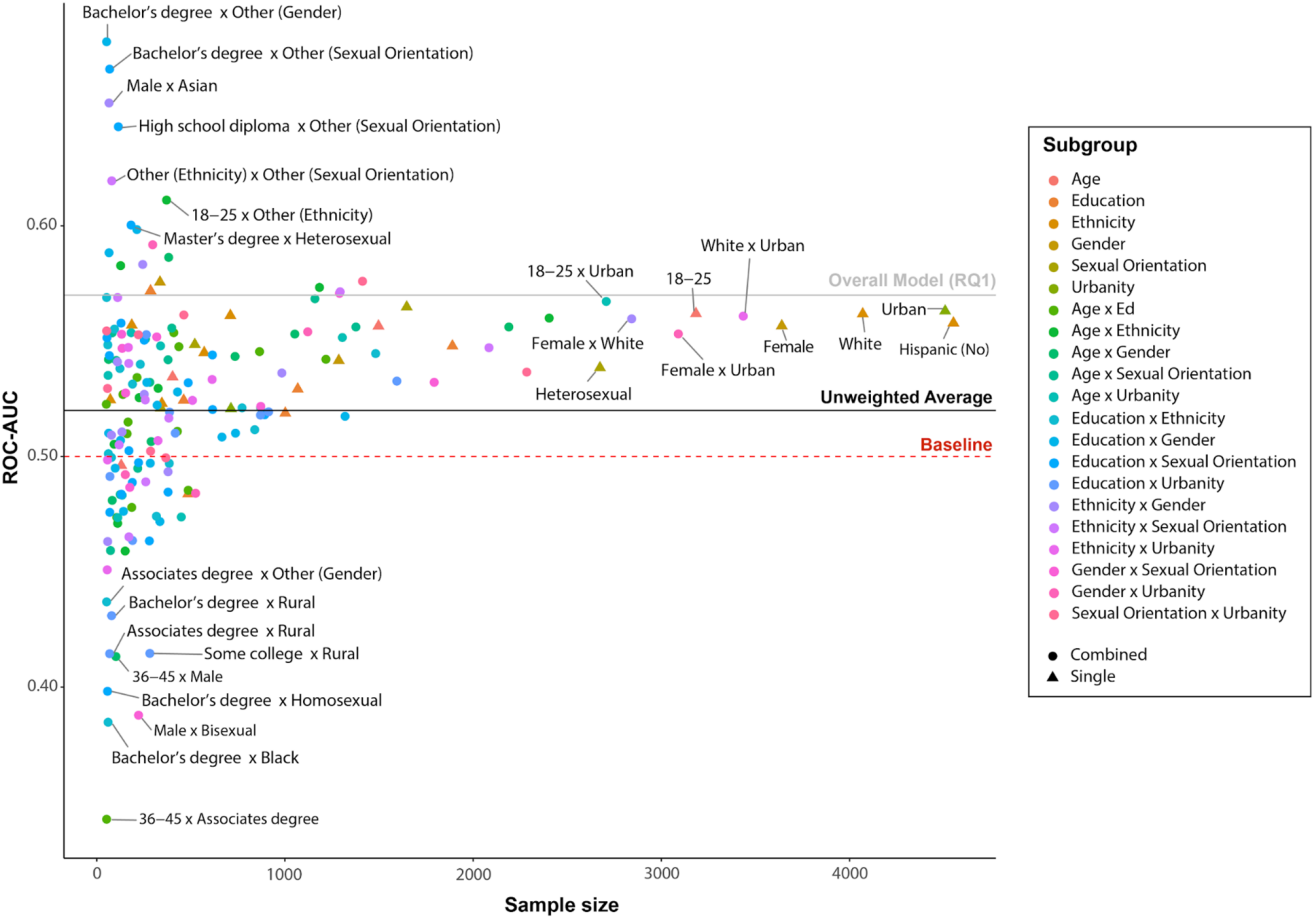
Out-of-sample predictive accuracies (AUC) of models trained and validated on the data of different socio-demographic subpopulation as a function of sample size. Shapes indicate whether the subpopulation is based on a single socio-demographic variable (circles) or a combination of two variables (triangles).

One may interpret our results as the curse of heterogeneous samples with a simple cure: Instead of training and validating predictive models on the general population, researchers first identify cohesive, homogeneous subsamples, and subsequently train and validate separate models for each of those subsamples. This practice is widely used in the field of machine learning^43,44^, because it allows predictive models to leverage the unique patterns between input and output variables observed for each subsample, thereby avoiding issues arising from generalization bias across groups (see^45^ for a review of subgroup effects in clinical trials). Translated to our particular prediction context, the logic behind such a procedure is simple: Students tend to resemble each other in terms of lifestyle, and they show little variation with regard to indicators such as geophysical context, socio-economic status, age, and education level^32^. This homogeneity might explain the higher predictive performance achieved in the student sample as opposed to the more heterogeneous sample. It is hence conceivable that different mobility patterns could be more predictive in homogeneous groups of individuals that can be subset from the overall population. For example, the same levels of physical activity and distance travelled might be considered little for a 22 year-old living in an urban area, while it might be considered a lot for a 78 year-old living in the country-side. To test whether the accuracy of general population predictions can be improved by dividing the overall sample into more homogeneous subsamples, we first split the U.S.-wide sample along the same socio-demographic lines as in the analyses for RQ2 (gender, age, education, urban/rural environment, sexual orientation and ethnicity, as well as their two-way intersections, e.g. age $\times$ gender). To increase the reliability of findings, we only included subgroups with at least 50 participants. For each of these groups, we trained and validated a model that was specifically fit for this subgroup. Similar to the analytic procedure for RQ1 and RQ2, all AUC scores are reported for cross-validated results (see Fig. 3). The resulting AUC scores range from 0.35 to 0.65 (mean = 0.52, SD = 0.05), demonstrating that splitting the heterogeneous sample into more homogeneous subsamples does not result in reliably higher predictive performance, and is nowhere near the accuracy observed in the on-campus sample even in the best case scenario. It is important to note that this is true for both small and large subpopulations. While the failure to improve accuracy for the smallest groups (left-hand side of Fig. 3) might be explained by the fact that there is too little training data for the model to pick up meaningful and robust relationships between mobility and depression, we also did not observe higher performance for the subsamples which have decent sample sizes.

**Figure 4:**
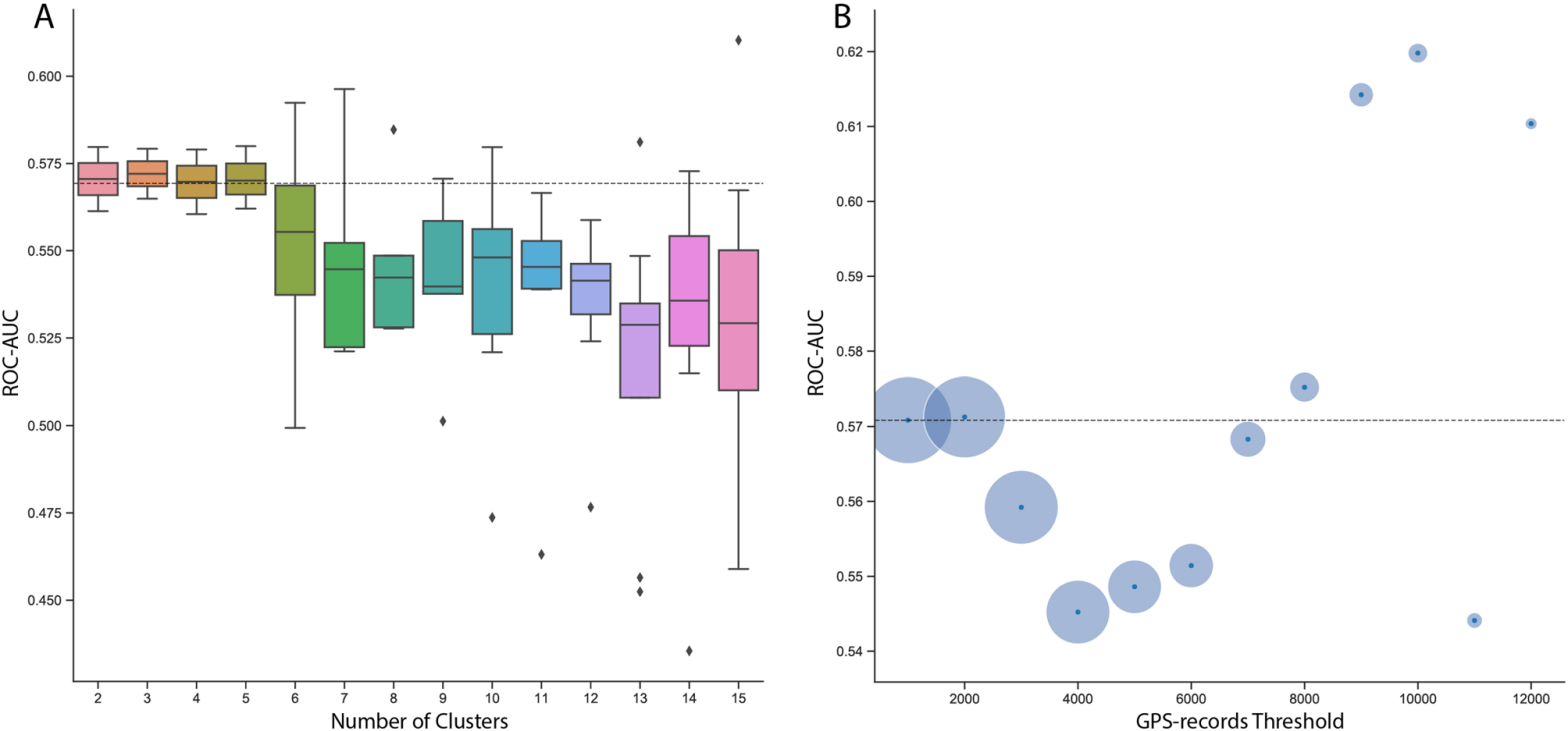
(**a**) Distribution of out-of-sample predictive performance (AUC) of clusters determined by K-means algorithm using different numbers of clusters. (**b**) Average out-of-sample predictive performance (AUC) of subsamples at various thresholds of GPS-records (e.g., 2000 records and higher). The size of the circle indicates the number of participants in each subsample. The dotted line indicates the average AUC score in the MindDoc sample.

In addition to dividing the sample into homogeneous subsamples based on socio-demographic characteristics, we also tested whether dividing the sample by similarity in mobility profiles could improve the accuracy of our predictive models. In fact, it is likely that students living on university campuses are more similar to each other not just in their socio-demographic composition but also in the way they engage with their physical environment. We therefore tested the impact of subsampling based on mobility features by testing predictive accuracy of models trained on clusters determined by mobility features. In order to ensure that the cluster assignment would not be affected by differences in mobility behaviors that in fact stem from differences in depression, we partialled out the effect of depression on all mobility metrics before conducting the cluster analysis. We then applied k-means clustering with varying numbers of clusters (2–15) on the residuals of the mobility features to test whether training and validating our model on subsamples that are similar in their mobility features could improve predictive accuracy. To prevent overfitting, we only included clusters with more than 100 participants. As Fig. 4A shows, we did not observe a meaningful increase in AUC. Splitting the sample into 2–5 clusters failed to improve accuracy, and splitting the sample into even more granular clusters, in fact, decreased the average accuracy (likely due to added noise resulting from small samples). In addition, we tested whether the predictive accuracy varied as a function of the threshold we set for the minimum number of GPS records per person. In Fig. 4B, we plot the AUC scores for various thresholds of GPS-records (i.e., only including participants with the number of GPS records above a certain threshold). We did not observe a reliable positive trend, suggesting that predictive accuracy in heterogeneous samples cannot be improved by focusing on users with large amounts of mobility data.

Taken together, these findings suggest that even dividing general population samples into more homogenous subsamples is less effective than one might hope and does not easily recover the drop in performance observed between the student and the general population samples. Notably, the findings reported in RQ3 are in line with the predictive performance outcomes obtained for the random forest XGBoost models in RQ1. Both models are able to represent non-linear effects and complex interactions of demographic categories and mobility patterns. Yet, we did not observe higher predictive performances of these models compared to the linear model. This lack in performance uptake in the non-linear models suggests that the inclusion of additional information about an individual’s socio-demographic characteristics does not increase the predictive value of mobility behaviors.

Collectively, the findings from RQ1–3 show that predicting depression from mobility is a difficult task. Even when offered the luxury of dissecting data across multiple combinations of demographic characteristics or along mobility clusters, predictive accuracy only increases for a handful of small groups. Moreover, the practice of clustering users in homogeneous groups, can result in vastly different levels of predictive performance between groups, raising concerns that the benefit of precise diagnostics is offered selectively.

## Discussion

Using technology to passively assess and monitor mental health issues, such as depression, holds great promise to improve both diagnostic processes, making it possible to detect symptoms early and efficiently for large populations. Mobility patterns captured by people’s smartphone sensors have been suggested as one promising avenue for such predictive technology^11,12,14,17,46^. However, our findings show that the same predictive modeling approach that leads to high prediction accuracy in a homogeneous student sample (validating prior research on the topic^12,17–22^), leads to predictive performance only slightly higher than chance in a socio-demographically heterogeneous U.S.-wide sample. Predictive performance was low across all socio-demographic groups, countering the possible explanation that the algorithm systematically performed well for some groups but poorly for others. While these findings suggest that it is generally difficult to predict depression in heterogenous samples, we found that training separate models for homogeneous subsamples did not substantially improve predictive performance either.

Taken together, our analyses provide convergent evidence that predicting depression from mobility behaviors among the general population is more difficult and arduous than what the previous results obtained in small, homogeneous student samples might suggest. One reason for why our attempt to recover some of the predictive performance by dividing our overall population into more homogeneous subsamples is that students who attend the same university are likely homogeneous in more than just their socio-demographic characteristics. Students are part of the same institutional environment, which means they are subject to the same set of norms and rules that prescribe and constrain behavior, including mobility behavior. Consequently, the variance observed in students’ mobility profiles is likely more meaningful and more predictive of mental health outcomes than that of the relatively broad socio-demographic subsamples used in our analyses.

Notably, this study focused on mobility patterns as the input feature of choice, because mobility data is easily available through GPS sensors. However, when considering different smartphone sensors, mobility might not be the most powerful when it comes to predicting depression in heterogeneous samples. Other data that can be passively collected using smartphone sensing includes smartphone usage, facial expression in images and videos, language used on social media, or sleep^47–51^. Such data may represent more reliable predictors of depression as behaviors captured from those data might fluctuate less across different geographic locations as well as socio-demographic groups and could therefore hold greater value in heterogeneous samples than GPS sensor data. For example, Eichstaedt and colleagues^47^ obtained an AUC of 0.69 when predicting depression from social media data, which falls in between the AUC achieved in the homogeneous student sample (N = 57; AUC = 0.82), and the AUC obtained in the U.S.-wide heterogeneous sample in this study (N = 5262; AUC = 0.57). However, while the sample of 686 patients might have been more heterogeneous that a cohort of college students, it is likely less heterogeneous than the general population. All patients were part of the same urban clinic are are therefore likely to share certain characteristics such as living in the same geographical area, using similar language, and possible sharing certain socio-demographic criteria.

The low predictive performance observed for the general population might also be due to the fact that using GPS data alone does not ascribe meaning to specific location patterns. As such, a person might be spending long hours at home because they are taking care of children, they are working from home, they caught the flu and are bedbound—or because they are severely depressed. Analyzing someone’s language use on social media (e.g. using many negatively valenced emotion words), or their phone usage patterns (e.g. spending hours passively scrolling through social media) might inherently provide more contextual meaning to the data. A combination of different data sources (e.g. location patterns in combination with phone usage) could help overcome this limitation.

The ability to capture mobility behaviors for large populations and map them against socio-demographic, psychological, and other behavioral variables holds great promise for advancing our understanding of mental health. However, our work cautions against generalizing findings based on small samples and making consequential decisions based on inferences about psychological traits and states from seemingly innocuous and prevalent patterns of human movement from sensors in smartphones and other digital devices. Policy makers need to take preventative action to protect individuals from such potentially biased and unfair decisions being made on the basis of their personal GPS data.

## Methods

### Samples and data collection

The data in this study was collected using the Android app MindDoc^34^. MindDoc allows users to continuously self-monitor their mood and depressive symptoms by responding to short surveys up to three times a day. The MindDoc screening tool consists of a pool of psychometric questions, 17 of which assess depressive symptoms (see Supplementary Table S1). For each symptom, users indicate whether they currently experience the symptom or not. If users indicate that a symptom is present, they are prompted to indicate how much the symptom currently burdens them, using a 4-point Likert-scale (see Supplementary Fig. S1). After 2 weeks of using the app a feedback report is generated automatically. If the user has not answered a sufficient number of surveys to generate the feedback report, the assessment period is reset and can be repeated.

#### Sample 1 (“Student Sample”)

Sample 1, the “Student Sample”, consisted of 112 students recruited at a large Northeastern University. We removed 18 students who did not complete the survey as well as 37 students with less than 1000 GPS-records. This led to a final sample of 57 participants with a total of 613,833 GPS records. For these participants, the mean number of GPS-records is 3350.98 (SD = 2327.90) and the mean number of days with more than 100 GPS-records is 12.81 days (SD = 2.21). In the final sample, 45.61% of participants were female, 71.93% were between the ages of 18–25, 21.05% between the ages of 26–35, and 7.02% were between the ages of 36–45. 80.70% of participants reported to be heterosexual, 7.02% bisexual and 5.26% homosexual. 66.67% of the sample were of Asian descent, 14.04% were White. 15.79% reported to be Hispanic. Data collection took place in the spring semester of 2020 and participants were paid $20 for their participation. Participants were asked to download the MindDoc app and use it for two weeks. Before the data collection period, all participants completed a short in-app on-boarding survey that captured demographic variables, including age, gender, education level. In addition, race, ethnicity, and sexual orientation were included as variables that have been shown to be linked to depression^52,53^ .

#### Sample 2 (“MindDoc User Sample”)

Sample 2, the “MindDoc User Sample”, consisted of 15,095 participants, who had downloaded the MindDoc app from the Google Play Store, and had volunteered to participate in a study through the app between December 2018 and February 2020. To provide a fair comparison of predictive accuracy across the two samples, we restricted the data to two weeks per participant. We removed 5533 participants located outside of the U.S., 3460 participants with less than 1000 GPS-records, and 840 participants with insufficient depression data. The final sample consisted of 5262 participants with a total number of 56,666,478 GPS-records. For these participants, the mean number of GPS-records is 4827.74 (SD = 2725.94) and the mean number of days with more than 100 GPS-records is 12.26 days (SD = 2.69). In the final sample, 69.14% of the participants were female, 60.50% were between 18–25 years old, 28.45% between 26–35 years old, and 11.05% were older. 77.35% of the participants were White, 3.53% were of Asian descent and 13.49% reported to be Hispanic. 50.82% indicated to be heterosexual, while 31.30% were bisexual and 6.48% were homosexual. 35.92% reported to have some college education, but no degree, 20.30% reported to have a high school diploma, 19.04% a Bachelor’s degree, and 8.78% less than high school. Sample 2 was further divided into subsamples according to a range of demographic criteria in order to analyze predictive performance in homogeneous samples (see this project’s OSF page for a detailed overview of the subsamples). Before the data collection period, all MindDoc users who volunteered to participate completed the same in-app onboarding questionnaire as the participants in Sample 1.

This study was approved by the ethics board of Columbia University in the City of New York (IRB Protocol No. AAAR9119). All research was performed in accordance with relevant guidelines and regulations, and all participants provided informed consent.

### Data preprocessing and feature engineering

The depression surveys obtained through the MindDoc app were scored according to the ICD-10 diagnostic rules. The ICD-10 distinguishes between three core symptoms (depressed mood, loss of interest and enjoyment, increased fatigue) and seven additional symptoms (reduced concentration and attention, reduced self-esteem and self-confidence, ideas of guilt and unworthiness, bleak and pessimistic views of the future, ideas or acts of self-harm or suicide, disturbed sleep, diminished appetite). Based on the number and type of symptoms present at a given point in time, the ICD-10 defines four different levels of depression severity ranging from “subclinical” to “severe”. In the present study, we used the definition of mild depression as a cutoff. That means individuals who showed at least two core symptoms and at least two additional symptoms during the two week assessment period were classified as “depressed”. For a detailed overview of the operationalizations and scoring rules of the ICD-10 depression criteria, please refer to Supplementary Table S2.

Sensing data was collected through the MindDoc app by way of event-based sampling. Every time the app registered that an individual changed their location, it recorded their latitude-longitude coordinates with a temporal resolution of 403.57 (SD = 250.72) GPS records per day and a spatial resolution of 24.92 meters (SD = 33.92). At the same time, accelerometer-based step count data was recorded through the Google Fit API^54^. A detailed overview of the preprocessing steps can be found in the Supplementary Methods.

From the raw GPS data, we computed 170 features which capture the extent to which participants’ moved around in their environment (e.g., total distance, max distance from home), how participants spent time in different locations (e.g., number of significant locations, the proportion of time spent at home), and the regularity of the participant’s movement within each day and across days (e.g., circadian movement, routine index). We applied the DBSCAN algorithm^55^ (eps = 30m, minPts = 3) to extract clusters and labeled the cluster where the user is most often located between 10:00 p.m. and 6:00 a.m. the next day as the participant’s home location. Thirty-eight mobility features were computed at the 14-day level, and 33 features were computed at the daily level. Daily values were aggregated using the mean, minimum, maximum, and standard deviation over the whole 14-day data collection period, resulting in an overall number of 170 GPS-based features. Step counts were extracted at a daily level and then aggregated over the 14-day period using the mean, minimum, maximum, standard deviation, sum, and the difference between weekdays and weekends, resulting in six additional step count features. A detailed overview of all features used in this study can be found in the Supplementary Methods. Prior to modelling, missing values were imputed using mean-imputation, and for the logistic regression classifier, all predictors were z-standardized.

### Modelling

We used three different machine learning algorithms—penalized logistic regression^36^, random forest^37^, and XGBoost^38^—in conjunction with nested cross-validation to predict depressive symptoms in the different samples (see Supplementary Methods). While logistic regression models capture linear relationships between the predictor and the outcome, random forest and gradient boost classifiers can capture more complex non-linear relationships and interactions among features. The inner cross-validation loop was used for hyperparameter tuning while the outer cross validation loop was used to assess generalized model performance. For each of the hyperparameter configurations performance was assessed using three-fold cross validation and the configuration with the highest area under the receiver operating characteristic curve (AUC-ROC) was applied to testing data in the outer loop. The outer loop used Monte-Carlo cross-validation with 100 stratified 80–20 splits in order to assess generalized model performance. Model performance was determined as the average AUC of predictions on the testing data. Furthermore, we conducted additional experiments and applied feature selection to limit the number of features to 50. We did not observe the predictive accuracy to be substantially higher after feature selection. A detailed overview of the hyperparameter spaces and search strategies used for each of the three algorithms can be found in the Supplementary Methods.

## Supplementary Information

Supplementary Information.

## Data Availability

Data (containing the features extracted from the raw data) and all code used for data preparation and analyses are shared on this project’s Open Science Framework (OSF) page ﻿at https://osf.io/pwvya/.
