# Supplementary material for: Depression predictions from GPS-based mobility do not generalize well to large demographically heterogeneous samples
Source: Sci Rep. 2021 Jul 7;11:14007. doi: 10.1038/s41598-021-93087-x (PMC8263566; doi:10.1038/s41598-021-93087-x)
Supplement: Supplementary file 1 — Supplementary Information. [file 41598_2021_93087_MOESM1_ESM.pdf]

Supplementary information for depression predictions from GPS-based mobility do not generalize well to large demographically heterogeneous samples

Sandrine R. Müller[1,+], Xi (Leslie) Chen[2,+], Heinrich Peters[3], Augustin Chaintreau[2], and Sandra C. Matz[3]

[1]Columbia University, Data Science Institute
[2]Columbia University, Computer Science Department
[3]Columbia University, Columbia Business School
[*]email: sm4658@columbia.edu
[+]these authors contributed equally to this work

Supplementary Figures S1: MindDoc Questionnaire User Interface

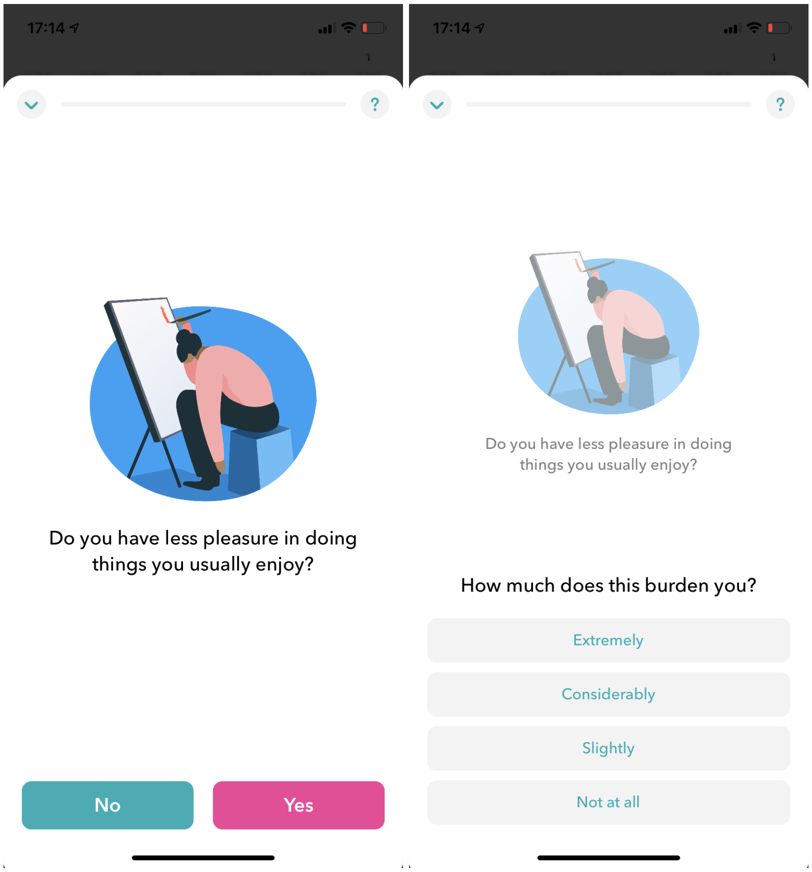

Figure 1. MindDoc app (https://mymoodpath.com/en/) depression screening (left) and follow-up (right) item. Included with permission from MindDoc Health GmbH.

Supplementary Figures S2: Number of Participants per State

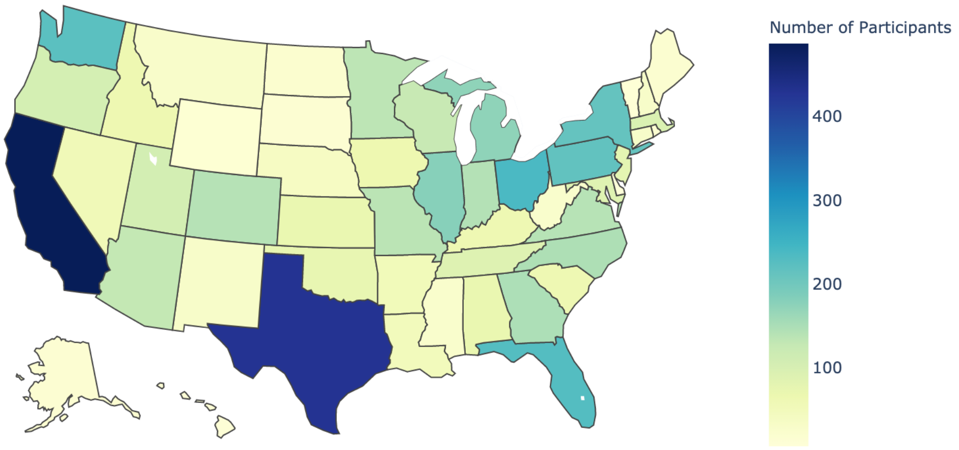

Figure 2. The number of participants per state in the MindDoc User Sample. The number of participants per state significantly correlates with the U.S.population per state (Pearson's $r(49) = 0.95, p < 0.001$). The map was generated using plotly 4.14.3 (`https://plotly.com/python/maps/`).

Supplementary Table S1. MindDoc depression screening.

Core symptoms	
1. Depressed mood	Are you feeling down and sad?<br>Are you feeling hopeless?
2. Loss of interest and enjoyment	Do you feel like you are not interested in anything right now?<br>Do you have less pleasure in doing things you usually enjoy?
3. Increased fatigability	Do you currently have considerably less energy?<br>Are your everyday tasks making you very tired currently?
Associated symptoms	
4. Reduced concentration and attention	Is it hard for you to make decisions currently?<br>Is it hard for you to concentrate currently?
5. Reduced self-esteem and self-confidence	Is your self-confidence clearly lower than usual?<br>Are you feeling up to your tasks?
6. Ideas of guilt and unworthiness	Are you blaming yourself currently?<br>Do you think you are worth less than others right now?
7. Bleak and pessimistic views of the future	Are you thinking that you will be doing well in the future?<br>Are you looking hopefully into the future?
8. Ideas or acts of self-harm or suicide	Are you thinking about death more often than usual?
9. Disturbed sleep	Did you sleep badly last night?
10. Diminished appetite	Do you have less or no appetite today?

Table 1. MindDoc depression screening questions according to ICD-10. To compute a depression score, each symptom must be assessed at least twice within a period of 14 days. A symptom is counted as potentially clinically significant if more than half of the assessments are above the cut-value of 2 on a 4-point severity scale (1-Not at all, 2-Slightly, 3-Considerably, 4-Extremely).

Supplementary Table S2. MindDoc diagnostic rules according to ICD-10.

Depression severity		Diagnostic criteria
0	None	No symptoms reported
1-2	Subclinical	At least 1 core or 1 additional ICD symptom
3	Mild	2 core and 2 additional ICD symptoms
4	Moderate	At least 2 core symptoms and sum of core and additional ICD symptoms ≥ 5
5	Severe	3 core and at least 4 additional ICD symptoms

Supplementary Table S3. Student and MindDoc Sample Descriptive.

Sample descriptive	Student	MindDoc
No. of participants	57	5262
Age 18-24, %	71.93	60.50
Age 26-35, %	21.05	21.05
Age >35, %	7.02	18.45
Female, %	45.61	69.14
Heterosexual, %	80.70	50.82
Homosexual, %	5.26	6.48
Bisexual, %	7.02	31.30
Asian, %	66.67	3.53
White, %	14.04	77.35
Hispanic, %	15.79	13.49
At least some college education, %	98.24	70.92
Mean No. of GPS records (SD)	3350.98 (2327.90)	4827.74 (2725.94)
Mean No. of Days (SD)	12.81 (2.21)	12.26 (2.69)

Supplementary Methods. Data Preprocessing

To associate participant's mobility traces to times of the day, we converted all timestamps from Coordinated Universal Time (UTC) to participant's local time. The participant's local time was determined as the time zone in which most of that participant's GPS records were located. Conflicts caused by switching between daylight saving time and standard time are resolved by deleting the earlier record within the overlapping periods.

Next, we converted GPS data into stationary periods. We filtered out GPS samples with a spatial resolution of more than 100 meters or that were recorded while moving. We determined whether a GPS sample was stationary or moving by computing its instantaneous speed. GPS records with an instantaneous speed below 1.4m/s - roughly the walking speed for adults - were defined as stationary. In order to extract clusters, we applied the DBSCAN algorithm[1] to the filtered GPS data. DBSCAN is particularly suitable in this case as it allows to identify clusters of varying shapes, does not require specifying the number of clusters ahead of time, and is robust to outliers[2]. We used Haversine distance as the distance function, and set epsilon to 30 meters and the minimal number of points to 3. We grouped consecutive GPS records if they belonged to the same cluster. We included all the grouped stationary periods that are longer than 10 minutes. In addition, we labeled the participant's home location as the place where the user is most often located between 10:00 p.m. and 6:00 a.m. the next day.

Supplementary Methods. Feature Extraction

We computed a comprehensive list of previously established mobility features[2–17]. We define a GPS sample as $L_i = (t_i, C_i)$, where t_i is the local time and C_i is the latitude-longitude pair. We denote a sequence of locations ordered by time as $L = \{L_1, ..., L_N\}$, where N is the number of GPS samples. Similarly, we denote a stationary period as $S_i = (t_i^a, t_i^d, C_i)$, where t_i^a is the time of arrival, t_i^d is the time of departure, and C_i is the latitude-longitude pair. A sequence of periods is denoted as $S = \{S_1, ..., S_M\}$, where M is the number of stationary periods. Based on the sequence of stationary periods, we compute the time spent at each cluster as $P_i = (d_i, C_i)$, where d_i is the duration of stay and C_i is the latitude-longitude pair. The list of clusters sorted in decreasing order of duration of stay is denoted as $P = \{P_1, ..., P_k\}$, where k is the number of clusters. The complete list of features are described below. Features are computed for both the daily as well as the 2-week aggregate level. Features indicated with * are only calculated at the 2-week level.

Number of Location Changes. We define this metric as the number of times the user switches to a different location cluster, which equals $M - 1$.

Location Variance. This metric is designed to measure the variability in the user's GPS locations. It is formally defined as the log of the combined variance of latitudes and longitudes

$$\log(\sigma_{lat}^2 + \sigma_{lon}^2)$$

where σ_{lat} is the standard deviation of the latitudes of GPS sample L and σ_{lon} is the standard deviation of the longitudes. As in[3], we used the logarithm to reduce the skewness of the distribution of location variance across users.

Number of GPS samples. This metric represents the number of GPS samples, which is N. Active users are expected to have more GPS samples.
Total Distance Covered. Formally, we define this metric as

$$\sum_{i=1}^{N-1} d(C_i, C_{i+1})$$

where $d(C_i, C_{i+1})$ is the Haversine distance[18] between two GPS coordinates.
Speed Mean and Variance. This metric measures the mean and variance of the instantaneous speed at each GPS location. For GPS location i from 1 to $N-1$, the instantaneous speed is defined as

$$\frac{d(C_i, C_{i+1})}{t_{i+1} - t_i}$$

Speed Quantile. This metric includes the 5, 10, 25, 50, 75, 90, and 95 percentiles of instantaneous speeds. It also includes the interquartile range (IQR) and skewness of instantaneous speeds.
Spatial Coverage. This metric measures the spatial area covered by the user, which is defined as the smallest Euclidian space that contains all location points. It is computed using the quickhull algorithm for convex hulls[19].
Transition Time This metric represents the fraction of GPS samples that were recorded in a moving state. It is formally defined as

$$\frac{\sum_{i=1}^{N-1} \mathbb{1}(C_i, C_{i+1}, t_i, t_{i+1})}{N-1}$$

where $\mathbb{1}(C_i, C_{i+1}, t_i, t_{i+1})$ is an indicator function which equals 1 if

$$\frac{d(C_i, C_{i+1})}{t_{i+1} - t_i} \geq 1.4\text{m/s}$$

Displacement Mean and Variance. For each location L_i with $1 \leq i \leq N-1$, we define displacement as $d(C_i, C_{i+1})$. The mean displacement is defined as

$$\bar{D} = \frac{1}{N-1} \sum_{i=1}^{N-1} d(C_i, C_{i+1})$$

And the variance of the displacements is defined as

$$\frac{1}{N-1} \sum_{i=1}^{N-1} (d(C_i, C_{i+1}) - \bar{D})^2$$

Maximum Distance from Home. Let C_h be the GPS coordinate of the home cluster. The maximum distance from home is then defined as

$$\max_{i \in \{1,\dots,N\}} d(C_i, C_h)$$

Maximum Distance. This metric measures the maximum distance between any two locations visited by the user. It is defined as

$$\max_{i,j \in \{1,\dots,N\}} d(C_i, C_j)$$

Number of Clusters. This metric measures the number of unique clusters visited by the user, which equals k. **Entropy.** This metric represents whether the user distributes his/her time uniformly across different clusters. Lower values indicate a user spent an unequal amount of time across clusters. If the user only spends time in one cluster, the entropy equals 0. Formally, this is defined as

$$-\sum_{i=1}^{k} d_i \log(d_i)$$

Normalized Entropy. Normalized entropy is defined such that entropy is invariant to the number of clusters. We divide entropy by the logarithm of the number of clusters such that the resulting value ranges between 0 and 1

$$\frac{-\sum_{i=1}^{k} d_i \log(d_i)}{\log(k)}$$

Raw Entropy. This metric measures entropy of the GPS samples before clustering. We divide the GPS coordinates into bins such that coordinates in the same bin are no more than 30 meters apart. Then we calculate the entropy of the number of GPS coordinates in each bin.
Percentage of Time Spent in Top-3 Clusters. For each of the top-3 clusters $i = 1,2,3$, it is defined as:

$$\frac{d_i}{\sum_{j=1}^{k} d_j}$$

Percentage of Time at Home. Let $P_h = (d_h, C_h)$ be the home cluster. Then, the percentage of time at home is defined as

$$\frac{d_h}{\sum_{i=1}^{k} d_k}$$

Percentage of Time in Cluster. The percentage of time in cluster is defined as

$$\frac{\sum_{i=1}^{k} d_k/3600}{x_{day} \cdot 24}$$

where x_{day} is the number of days and d_k is in the unit of seconds.
Average Time at each Location. This metric measures the average time spent at each cluster

$$\frac{\sum_{i=1}^{k} d_k}{k}$$

Displacement Entropy. This feature measures whether the displacement of a user is evenly distributed across each hour. If the user covers a similar amount of distance across all hours of the day, the displacement entropy will be high. We create 21 distance bins ranging from 200m to 4000m (and more). Then, we compute the distance covered for each hour of the day and place the hour into the corresponding distance bin. The displacement entropy is defined as the entropy of the vector formed by the distance bin.
The Radius of Gyration. As in[5], the metric is defined as the deviation from the centroid of the clusters visited - which is

$$\bar{C} = \frac{\sum_{i=1}^{k} C_i}{k}$$

weighted by the time spent at each cluster:

$$\frac{\sum_{j=1}^{k} d_j \cdot d(C_j, \bar{C})}{\sum_{i=1}^{k} d_i}$$

where $d(C_j, \bar{C})$ is the Haversine distance between C_j and the centroid.
Circadian Movement*. We adopt the method from[3] to measure the extent to which a user's location sequences follow a 24-hour rhythm. If the user follows a similar routine each day, the circadian movement will be high.
Number of Days*. This metric measures the number of days the user has GPS samples for.
Routine Index*. As in[5], we define the routine index as an abstract representation of a user's mobility trace on each day. We compute the maximum, minimum, and standard deviation of daily differences in routine index for every pair of days.

Supplementary Methods. Hyper-parameter search space

For the logistic regression, we employed grid search to select the best hyper-parameters and evaluated each hyper-parameter using 3-fold cross-validation with AUC scoring function. The search space is the following:

- Penalty: L1, L2
- C: 1e-5, 1e-4,..,100,1000

For random forest, we we used randomized search to select the best hyper-parameters and evaluated each of the 50 randomly selected hyper-parameters using 3-fold cross-validation with AUC scoring function. The search space is the following:

- Bootstrap: True, False
- Maximum depth: 1 to 10 with a step size of 1

- Minimum samples leaf: 1,2,4
- Minimum samples split: 2,5,10
- Number of estimators: 100 to 500 with a step of 100

For XGboost, we used randomized search to select the best hyper-parameters and evaluated each of the 50 randomly selected hyper-parameters using 3-fold cross-validation with AUC scoring function. The search space is the following:

- Number of estimators: 100 to 400 with a step size of 100
- Maximum depth: 2 to 10 with a step size of 1
- Learning rate: 0.001, 0.01, 0.1, 0.2, 0.3, 0.4
- Gamma: 1 to 10 with a step size of 1

References

1. Ester, M., Kriegel, H.-P., Sander, J., Xu, X. *et al.* A density-based algorithm for discovering clusters in large spatial databases with noise. In *KDD*, vol. 96, 226–231 (1996).
2. Farhan, A. A. *et al.* Behavior vs. introspection: refining prediction of clinical depression via smartphone sensing data. In *2016 IEEE Wireless Health (WH)*, 1–8 (IEEE, 2016).
3. Saeb, S. *et al.* Mobile phone sensor correlates of depressive symptom severity in daily-life behavior: an exploratory study. *J. Med. Internet Res.* **17**, e175 (2015).
4. Xu, X. *et al.* Leveraging routine behavior and contextually-filtered features for depression detection among college students. *Proc. ACM on Interactive, Mobile, Wearable Ubiquitous Technol.* **3**, 1–33 (2019).
5. Canzian, L. & Musolesi, M. Trajectories of depression: unobtrusive monitoring of depressive states by means of smartphone mobility traces analysis. In *Proceedings of the 2015 ACM International Joint Conference on Pervasive and Ubiquitous Computing*, 1293–1304 (2015).
6. Chen, R. *et al.* Developing measures of cognitive impairment in the real world from consumer-grade multimodal sensor streams. In *Proceedings of the 25th ACM SIGKDD International Conference on Knowledge Discovery & Data Mining*, 2145–2155 (2019).
7. Wang, R. *et al.* Tracking depression dynamics in college students using mobile phone and wearable sensing. *Proc. ACM on Interactive, Mobile, Wearable Ubiquitous Technol.* **2**, 1–26 (2018).
8. Saeb, S., Lattie, E. G., Schueller, S. M., Kording, K. P. & Mohr, D. C. The relationship between mobile phone location sensor data and depressive symptom severity. *PeerJ* **4**, e2537 (2016).
9. Huang, Y. *et al.* Assessing social anxiety using gps trajectories and point-of-interest data. In *Proceedings of the 2016 ACM International Joint Conference on Pervasive and Ubiquitous Computing*, 898–903 (2016).
10. Chow, P. I. *et al.* Using mobile sensing to test clinical models of depression, social anxiety, state affect, and social isolation among college students. *J. medical Internet research* **19**, e62 (2017).
11. Saeb, S., Lattie, E. G., Kording, K. P. & Mohr, D. C. Mobile phone detection of semantic location and its relationship to depression and anxiety. *JMIR mHealth uHealth* **5**, e112 (2017).
12. Wang, W. *et al.* Sensing behavioral change over time: Using within-person variability features from mobile sensing to predict personality traits. *Proc. ACM on Interactive, Mobile, Wearable Ubiquitous Technol.* **2**, 1–21 (2018).
13. Lu, J. *et al.* Joint modeling of heterogeneous sensing data for depression assessment via multi-task learning. *Proc. ACM on Interactive, Mobile, Wearable Ubiquitous Technol.* **2**, 1–21 (2018).
14. Ben-Zeev, D., Scherer, E. A., Wang, R., Xie, H. & Campbell, A. T. Next-generation psychiatric assessment: Using smartphone sensors to monitor behavior and mental health. *Psychiatr. Rehabil. J.* **38**, 218 (2015).
15. Gruenerbl, A. *et al.* Using smart phone mobility traces for the diagnosis of depressive and manic episodes in bipolar patients. In *Proceedings of the 5th Augmented Human International Conference*, 1–8 (2014).
16. Palmius, N. *et al.* Detecting bipolar depression from geographic location data. *IEEE Transactions on Biomed. Eng.* **64**, 1761–1771 (2016).

17. Wang, R. *et al.* Crosscheck: toward passive sensing and detection of mental health changes in people with schizophrenia. In *Proceedings of the 2016 ACM International Joint Conference on Pervasive and Ubiquitous Computing*, 886–897 (2016).

18. Sinnott, R. W. Virtues of the haversine. *Sky Telesc.* **68**, 158 (1984).

19. Barber, C. B., Dobkin, D. P. & Huhdanpaa, H. The quickhull algorithm for convex hulls. *ACM Transactions on Math. Softw. (TOMS)* **22**, 469–483 (1996).
